# Unlocking the Hidden Potential of Cancer Therapy Targeting Lysine Succinylation

**DOI:** 10.7150/jca.105849

**Published:** 2025-01-01

**Authors:** Zhuomeng Zheng, Peiyao Xiao, Jiale Kuang, Zhiyu Wang, Xinyu Wang, Da Huang, Yuxuan Guo, Li Zhou, Yiyuan Yang, Siyu Ding, Chanjuan Zheng, Yian Wang, Shujun Fu, Xiyun Deng

**Affiliations:** Key Laboratory of Translational Cancer Stem Cell Research, Department of Pathophysiology, School of Basic Medical Sciences, Hunan Normal University, Changsha, Hunan 410013, China.

**Keywords:** Lysine Succinylation, Cancer Therapy, Metabolic Reprogramming, Gene Expression, Oncogenic Signaling Pathways, Tumor Immune Microenvironment

## Abstract

Lysine succinylation is an emerging post-translational modification of proteins. It involves the addition of the succinyl group to lysine residues of target proteins through both enzymatic and non-enzymatic pathways. This modification can alter the structure of the target protein, which, in turn, impacts protein activity and function and is involved in a wide range of diseases. In the field of cancer biology, lysine succinylation has been shown to exert a substantial influence on metabolic reprogramming of tumor cells, regulation of gene expression, and activation of oncogenic signaling pathways. Furthermore, lysine succinylation modulates the activity of immune cells, thereby affecting the immune evasion of tumor cells. Notably, researchers are currently developing inhibitors and activators of lysine succinylation which can inhibit tumor cell proliferation, migration, and metastasis, with potential usefulness in future clinical practice. This article provides an overview of the biological functions of lysine succinylation in cancer and its potential applications in cancer treatment, offering a novel perspective for future cancer management.

## 1. Introduction

In 2022, there have been nearly 20 million new cases of malignant tumors worldwide, with an estimated 9.7 million deaths 1. Malignant cancers, such as lung cancer, colorectal cancer, liver cancer, and breast cancer, are among the primary causes of mortality, which has drawn widespread attention. As a significant threat to human health, the occurrence and development of malignant tumors involve intricate and finely regulated molecular mechanisms 2. Therefore, understanding the pathophysiological and biochemical mechanisms of biomolecule regulation is crucial for accurate diagnosis and effective treatment of cancer 3.

Post-translational modifications (PTMs) represent a crucial mechanism for the functional regulation of proteins within cells. By adding specific modifications, such as phosphorylation, glycosylation, acetylation, ubiquitination, crotonylation, and succinylation, to the amino acid residues of proteins, PTMs can finely regulate the activity, stability, subcellular localization, and interactions to adapt to the needs of complex life activities of organisms 4. In the context of malignant tumor development, PTMs are involved in several hallmarks of cancer, including sustaining proliferative signaling, evading growth suppressors, resisting cell death, enabling replicative immortality, activating invasion, inducing angiogenesis and metastasis, etc 5. Due to the pivotal role of PTMs in malignant tumor development, studies targeting intracellular regulatory enzymes with protein-modifying functions have attracted widespread attention in the field of new drug development.

Lysine succinylation is a conversed PTM identified by Zhang *et al.* in 2011 6. This modification is reversible, inherently dynamic, and evolutionarily conserved. Lysine succinylation refers to adding a succinyl group to a lysine residue. The introduction of a succinyl group not only neutralizes the intrinsic positive charge of the lysine residue but also introduces a sizeable change in molecular weight. The succinyl group has a molecular weight of approximately 100 Da, which is considerably larger than the molecular weights of the other modifying groups, such as acetyl and methyl groups (42 Da and 14 Da, respectively). Such a change in charge and the introduction of a larger chemical group is more likely to cause significant changes in the protein conformation, which, in turn, affects its interactions with other molecules, thus playing a vital role in the regulation of protein activity and function 7. A variety of cancers are associated with dysregulation of lysine succinylation, including breast cancer, renal cancer, gastrointestinal tumors, and prostate cancer 8-10.

With the development of high-resolution mass spectrometry and affinity enrichment techniques, scientists continue to discover more lysine succinylation sites and sequence variants in cancer. The abnormal succinylation of lysine residues has emerged as a multifaceted participant, demonstrating varied roles in diverse cancer types. Lysine succinylation has been found to promote cell metabolism, regulate gene expression, enhance signaling, and suppress immune responses in tumor cells. To further illustrate the complex relationship between succinylation modification and tumors and the current research on succinylation-targeting drugs, we have conducted a comprehensive review of the existing research articles regarding the roles of succinylation in cancer development, trying to provide some useful information for the exploration of more effective drug targets.

## 2. Regulation of Lysine Succinylation/desuccinylation in Cancer

**Figure 1:**
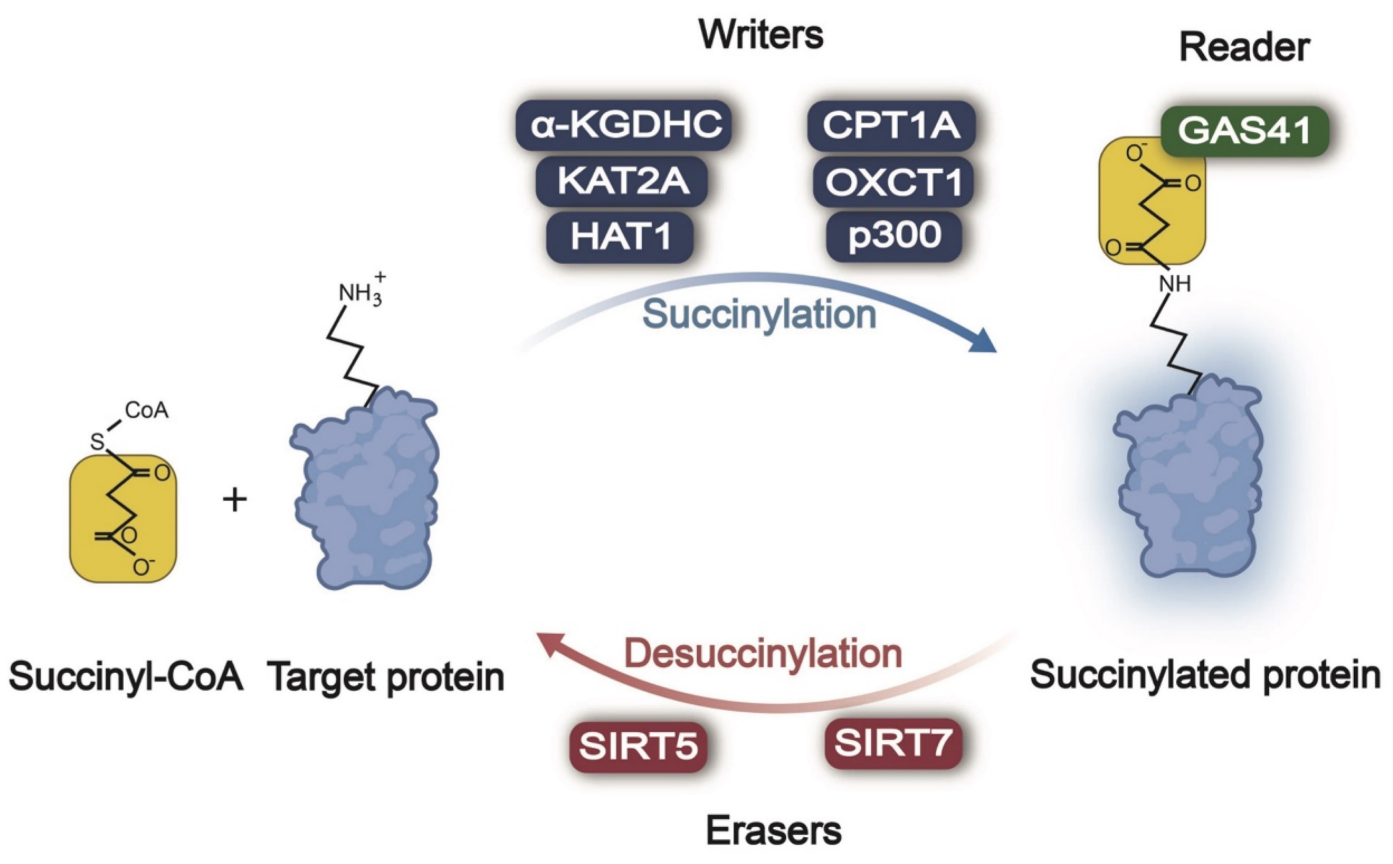
** Enzymatic regulation of lysine succinylation/desuccinylation in cancer.** Across different types of cancer, α-KGDHC, KAT2A, HAT1, CPT1A, OXCT1A, and p300 act as writers, promoting the succinylation of the lysine residue. SIRT5/7 serve as erasers, causing the desuccinylation of the lysine residue. GAS41 behaves as a reader, recognizing lysine succinylation of the target protein and transducing the succinylation signal. Succinyl-CoA: succinyl coenzyme A; α-KGDHC: α-ketoglutarate dehydrogenase complex; KAT2A: lysine acetyltransferase 2A; HAT1: histone acetyltransferase 1; CPT1A: carnitine palmitoyltransferase 1A; OXCT1: 3-oxoacid CoA-transferase 1; SIRT5: sirtuin 5; SIRT7: sirtuin 7; GAS41: glioma amplified sequence 41.

In the processes of protein succinylation and desuccinylation (**Figure 1**), succinylases act as "writers" responsible for adding a succinyl group to lysine residues of the target protein, while desuccinylases serve as "erasers" tasked with removing these modifications. Subsequently, these modifications are recognized by "reader(s)" that regulate the function of proteins accordingly. The mechanism and function of succinylation-related enzymes in tumors are summarized in **Table 1**.

### 2.1 Enzymatic Regulation of Lysine Succinylation in Cancer

As "writers", succinylases play a significant role in cancer development and progression by regulating various cellular processes. The α-ketoglutarate dehydrogenase complex (α-KGDHC) represents a crucial multi-enzyme complex within the tricarboxylic acid (TCA) cycle, composed of three main subunits: E1k (α-ketoglutarate dehydrogenase, EC 1.2.4.2), E2k (dihydrolipoyl succinyltransferase, EC 2.3.1.61), and E3k (dihydrolipoyl dehydrogenase, EC 1.8.1.4). α-KGDHC can provide succinyl coenzyme A (succinyl-CoA) to succinyltransferase lysine acetyltransferase 2A (KAT2A), promoting the succinylation of histone H3, which leads to tumor cell proliferation and tumor formation 11. It is noteworthy that the efficiency of the α-KGDHC complex is dependent on the E2k subunit 12. If the α-KGDHC complex is responsible for providing succinyl groups within the organism, it can be deduced that most biochemical pathways involving succylation are likely regulated by the α-KGDHC complex.

KAT2A, also known as GCN5, was initially identified as an acetyltransferase with the ability to bind to acetyl-CoA and transfer its acetyl group to histones 13. Subsequently, KAT2A was identified as a succinyltransferase capable of inducing histone H3 succinylation on lysine 79 (H3K79) through its interaction with α-KGDHC, promoting the proliferation and development of glioblastoma 11. Tong *et al.* demonstrated that KAT2A is highly expressed in human pancreatic ductal adenocarcinoma and enhances β-catenin expression to promote glycolysis in pancreatic ductal adenocarcinoma cells undergoing epithelial-to-mesenchymal transition (EMT). This facilitates cell proliferation, migration, and invasion of cancer 14.

Histone acetyltransferase 1 (HAT1) was initially characterized as an acetyltransferase that regulates the acetylation of histones and non-histone proteins 15. In malignant tumors, including liver cancer, pancreatic cancer, and cholangiocarcinoma, the expression level of HAT1 was significantly elevated, which suggested that HAT1 was closely related to tumorigenesis 16. HAT1 activates gene expression by facilitating the succinylation of histone H3 at K122 16. Concurrently, HAT1 also enhances the glycolytic process by promoting the succinylation of phosphoglycerate mutase 1 (PGAM1) at K99 17. Through these two biological pathways, HAT1 plays a pivotal regulatory role in the initiation and progression of the aforementioned cancers.

Carnitine palmitoyltransferase 1A (CPT1A) is the rate-limiting enzyme in fatty acid oxidation, which transports fatty acids to the mitochondrial matrix for β-oxidation. CPT1A, rather than relying on its typical CPTase activity, can regulate cellular metabolism by succinylating substrate proteins. Moreover, this enzyme exhibits lysine succinyltransferase (LSTase) activity both *in vitro* and *in vivo* 18. In gastric cancer, CPT1A succinylates LDHA at K222, thereby inhibiting its degradation and promoting the proliferation and invasion of gastric cancer cells 19. In ovarian cancer, CPT1A promoted the succinylation of mitochondrial fission factor (MFF) at K302 to regulate mitochondrial fission and function and promoted the growth and proliferation of ovarian cancer cells 20.

3-Oxoacid CoA-transferase 1 (OXCT1), a pivotal rate-limiting enzyme in ketone body catabolism, facilitates the transfer of the coenzyme A moiety from succinyl-CoA to acetoacetate, resulting in the formation of acetoacetyl coenzyme A 21. Serine β-lactamase-like protein (Lactb), a tumor suppressor, regulates mitochondrial lipid metabolism and complex signaling pathways through protein hydrolase activity. OXCT1 inhibited the protein hydrolase activity of Lactb and enhanced mitochondrial respiration by promoting the succinylation of Lactb at K284, which further promotes the progression of hepatocellular carcinoma 22, 23.

P300 functions as a histone acetyltransferase and a transcriptional cofactor 24. It has recently been demonstrated that p300 proteins can catalyze both acetylation and succinylation. Shahidian and colleagues found that p300 could succinylate histone H3 at K122 25. Ma and coworkers demonstrated that p300 succinylated pivotal cytoplasmic glycolytic enzymes (e.g., PGK1, PKM, PFKL, and ALDOA) to facilitate glycolysis in lung adenocarcinoma (LUAD) 26.

The regulation of the expression and activity of succinyltransferases, including α-KGDHC, KAT2A, HAT1, CPT1A, OXCT1, and p300, enables cancer cells to alter the function and stability of proteins, which, in turn, affects cell proliferation, invasion, and metastasis. Further research into the regulation of enzymatic lysine succinylation in cancer cells may provide new targets for cancer therapy.

### 2.2 Enzymatic Regulation of Lysine Desuccinylation in Cancer

Protein succinylation is a reversible PTM, and in the process of in-depth research on this modification, various desuccinylases that play a negative regulatory role have been successively discovered. In 2013, Colak *et al.* identified CobB, a lysine deacetylase with structural and functional similarities to SIRT2, as the first prokaryotic desuccinylase 27. In eukaryotes, three desuccinylases have been identified: sirtuin 5 (SIRT5), sirtuin 7 (SIRT7), and histone deacetylase1/2/3 (HDAC1/2/3) 28-30. In tumors, the aberrant activity of these desuccinylases may be associated with critical biological processes, including metabolic reprogramming, proliferation, apoptosis resistance, and tumor microenvironment adaptation.

SIRT5 has been identified as an NAD^+^-dependent protein lysine deacetylase with a primary location in the mitochondrion. Despite its limited deacetylation capacity, SIRT5 has been shown to possess desuccinylation, deglutaminylation, and depropanoylation capabilities. Its regulatory role extends to proteins involved in diverse metabolic pathways, including glycolysis, the TCA cycle, fatty acid oxidation, the electron transport chain, ketone body production, nitrogenous waste disposal, and reactive oxygen species (ROS) detoxification 31. Therefore, SIRT5 dysregulation can affect tumor development in several ways. For example, SIRT5 regulates glutamine metabolism by desuccinylating glutaminase (GLS), which has been demonstrated to promote breast cancer development 32. Additionally, SIRT5 has been shown to promote clear cell renal cell carcinoma by inhibiting lysine succinylation of succinate dehydrogenase complex subunit A (SDHA) 10. Nevertheless, SIRT5 has been demonstrated to exert an inhibitory effect on the development of certain tumors. Chen *et al.* showed that SIRT5-mediated desuccinylation inhibited hepatocellular carcinoma development by inhibiting acyl-CoA oxidase 1(ACOX1) 33. Lu and coworkers demonstrated that SIRT5 inhibited the growth and migration of gastric cancer cells by interfering with mitochondrial function and redox state through the desuccinylation of 2-oxoglutarate dehydrogenase (OGDH) and the inhibition of OGDH complex activity 34. Lin and colleagues demonstrated that SIRT5 bound to copper/zinc superoxide dismutase (SOD1), desuccinylated SOD1, and activated SOD1 to eliminate ROS and inhibited lung cancer cell growth 35.

SIRT7 is another desuccinylase with established functionality. It has been demonstrated that SIRT7 can repair broken DNA double strands by promoting chromatin condensation 28. Furthermore, SIRT7 has been shown to mediate the desuccinylation of protein arginine methyltransferase 5 (PRMT5) at K387, which has been linked to the promotion of lipid metabolism and, consequently, cancer cell proliferation, migration, and invasion 36. Furthermore, H3 desuccinylation at K122 by SIRT7 affected hepatitis B virus (HBV) transcription and replication 37. HBV is a key factor in causing hepatocellular carcinoma, as their persistent infection leads to chronic inflammation and liver cell damage, which promotes the development and progression of hepatocellular carcinoma 38. Despite being less extensively studied than SIRT5, SIRT7's potential enzymatic activity and cellular function may play a significant role in cancer metabolism.

HDAC1/2/3 play a pivotal role in regulating histone succinylation levels, particularly during histone desuccinylation in promoter regions 30. Nevertheless, the current studies lack direct evidence linking HDAC-mediated desuccinylation to tumors. This is an area that requires further research.

Taken together, desuccinylases play a significant role in cancer development. A comprehensive understanding of these enzymes is essential for developing effective therapeutic strategies against cancer. Future research efforts will continue to investigate the specific roles of desuccinylases in cancer metabolism to introduce innovative therapeutic approaches and strategies to cancer treatment.

### 2.3 Regulation of Lysine Succinylation by Other Factors in Cancer

Succinyl-CoA is the primary donor of succinyl groups in the lysine succinylation reaction and can be directly involved in the succinylation process as a substrate. Therefore, succinyl-CoA concentration has an important impact on the degree of lysine succinylation 39. Succinyl-CoA is produced by various pathways, mainly including the TCA cycle, fatty acid metabolism, and amino acid metabolism. In the TCA cycle, α-KGDHC catalyzes the conversion of α-ketoglutarate to succinyl-CoA, producing NADH and CO_2_. The fatty acid oxidation pathway in the peroxisome also generates succinyl-CoA, which is subsequently exported to the cytoplasm as succinyl carnitine to perform physiological roles 40. A number of amino acids, including valine, isoleucine, and methionine, can also be metabolized to produce succinyl-CoA 40. In addition, succinyl-CoA can be catalyzed to succinate by succinyl-CoA synthetase. In the presence of succinate dehydrogenase (SDH), succinate is oxidized to fumarate. Inhibition of the critical enzyme SDH leads to a succinyl-CoA build-up, causing hyper-succinylation and contributing to cancer development 41.

In tumors, pH value can also affect the occurrence of lysine succinylation. In 2013, Wagner and coworkers found that pH can drive lysine succinylation occurrence in mitochondria in non-enzymatic conditions. And the higher the pH, the greater the degree of lysine succinylation 42. In addition to this, pH also affects lysine succinylation recognition. Glioma amplified sequence 41 (GAS41) is a reader for succinylated proteins 43. At a low pH, the affinity between GAS41 and H3K122succ was enhanced. When the pH is increased to 7.4, the affinity between GAS41 and H3K122succ was weakened 43. This suggests that GAS41 reads succinylation modification in a pH-dependent manner 44.

## 3. Impact of Lysine Succinylation on Tumor Development

As mentioned above, since its discovery, protein succinylation has been shown to play roles in various aspects of tumor development 45-47. Firstly, in metabolic reprogramming, lysine succinylation regulates energy metabolism and biosynthetic pathways in tumor cells, affecting cellular uptake and utilization of nutrients, thereby supporting the rapid proliferation and survival of tumor cells. Secondly, as an epigenetic modification, lysine succinylation can adjust gene expression patterns at the molecular level. This is a process that involves both alterations to histone conformation and regulation of transcription factor activity. Thirdly, lysine succinylation can promote the proliferation, migration, and invasion of tumor cells by altering the function and stability of key signaling molecules, exacerbating the malignant process of cancer. Finally, in the immune microenvironment, lysine succinylation shapes a local environment conducive to tumor growth and metastasis by influencing the activity of immune cells.

### 3.1 Involvement of Lysine Succinylation in Metabolic Reprogramming

**Figure 2:**
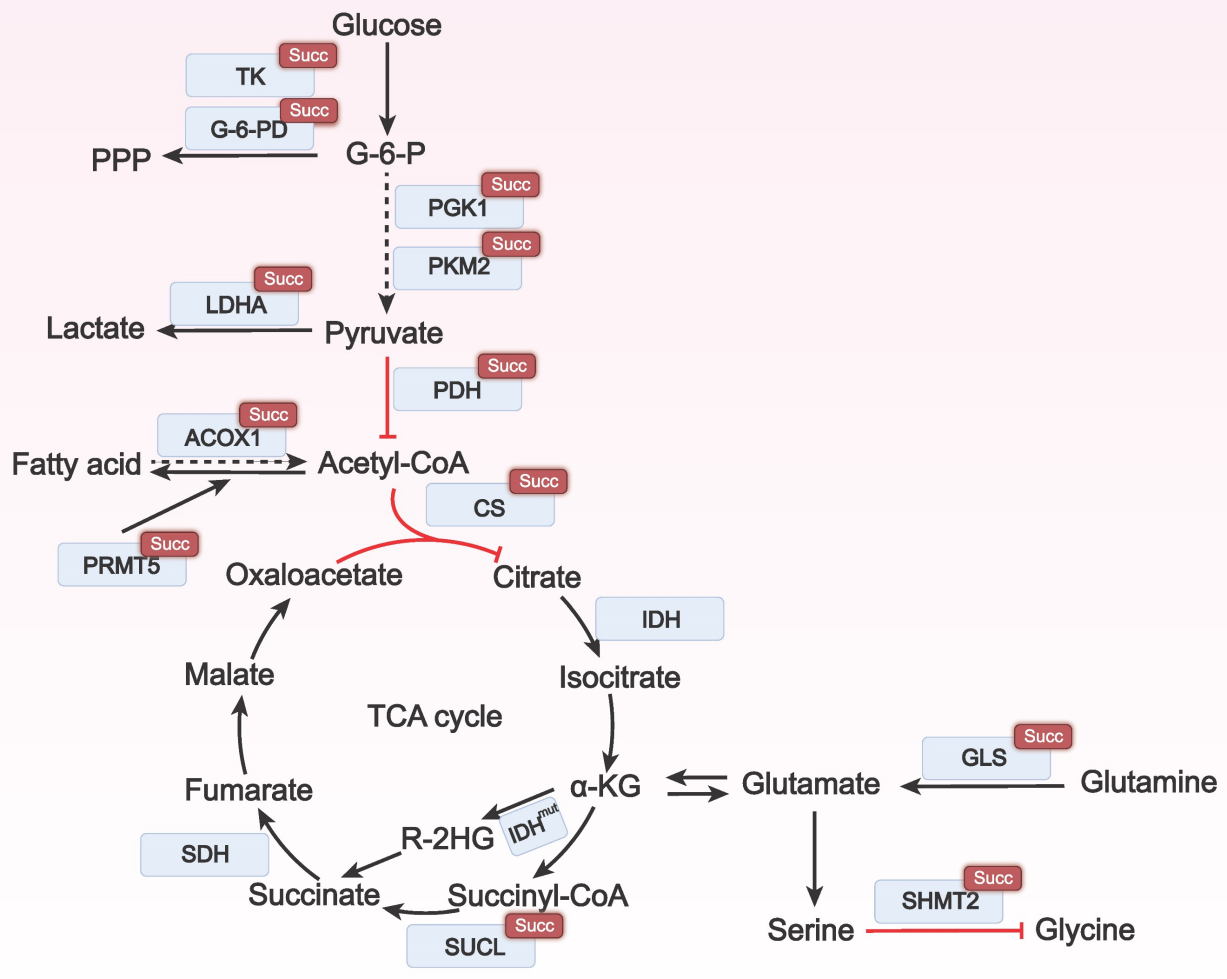
** The role of lysine succinylation in metabolic reprogramming.** In various cancers, succinylation can regulate multiple metabolic enzymes in glycolysis, amino acid metabolism, and lipid metabolism, thereby affecting the occurrence and development of tumors. Succ: succinylation; TK: transketolase; G-6PD: glucose-6-phosphate dehydrogenase; PGK1: phosphoglycerate kinase 1; PKM2: pyruvate kinase M2; LDHA: lactate dehydrogenase A; PDH: pyruvate dehydrogenase; ACOX1: acyl-CoA oxidase 1; PRMT5: protein arginine methyltransferase 5; Acetyl-CoA: acetyl coenzyme A; CS: citrate synthase; IDH: isocitrate dehydrogenase; R-2HG: R-2-hydroxyglutarate; succinyl-CoA: succinyl coenzyme A; SUCL: succinyl-CoA ligase; SDH: succinate dehydrogenase; GLS: glutaminase; SHMT2: serine hydroxymethyltransferase 2; TCA cycle: tricarboxylic acid cycle.

Metabolic reprogramming is a process required for tumor cells to adapt to the harsh microenvironment and maintain their survival and proliferation. Succinylation regulates the activity of key metabolic enzymes and affects the utilization of nutrients such as glucose, lipids, and amino acids to meet the energy and raw material requirements of tumor cells for growth (**Figure 2**).

#### 3.1.1 Lysine Succinylation in Glucose Metabolism

In normal cells, glycolysis, the TCA cycle, and the pentose phosphate pathway (PPP) are three key intracellular glucose metabolism pathways that together provide cells with energy and biosynthetic precursors. In tumor cells, these metabolic pathways can also support tumor cell survival, proliferation, and metastasis 48. During glycolysis, there are multiple metabolic enzymes involved, such as phosphoglycerate kinase 1 (PGK1), pyruvate kinase M2 (PKM2), and lactate dehydrogenase A (LDHA). These enzymes can be regulated by succinylation modifications, thereby affecting their activities or functions and participating in tumorigenesis and development 49. Some studies have found that during glycolysis, inhibiting the succinylation enzyme p300 leads to a decrease in the succinylation level of multiple glycolytic enzymes, especially PGK1 23. This significantly hinders cellular glycolysis and lactate production. Specifically, KAT2A can enhance the glycolysis process by promoting the succinylation of the critical glycolytic enzyme PKM2 at K475, thereby facilitating the growth of gastric cancer cells 50. In tumor cells, succinylation of PKM2 at K433 stabilizes voltage-dependent anion channel 3 (VDAC3), leading to increased mitochondrial permeability and ATP production, which supports cell survival during glucose starvation 51. Carnitine palmitoyltransferase 1A (CPT1A), by influencing LDHA at K222, increases its succinylation level, inhibiting LDHA degradation, thus enhancing glycolysis and promoting the invasion and proliferation of gastric cancer 19. In prostate cancer, succinylation of LDHA at K118 significantly enhances the migration and invasion capabilities of tumor cells 52. Pyruvate dehydrogenase (PDH) is the enzyme connecting glycolysis and the TCA cycle. It converts pyruvate, the final product of glycolysis, into acetyl-CoA, the starting substrate for the TCA cycle. Downregulation of SIRT5 in clear cell renal cell carcinoma leads to hyper-succinylation of PDH, reducing PDH activity, which accelerates the Warburg effect and induces tumorigenesis and progression 53.

Recent research evidence has revealed that specific cancer cells heavily rely on the TCA cycle for energy production, redox balance maintenance, and biosynthesis of biomacromolecules 54. Succinyl-CoA is a crucial intermediate in the TCA cycle, vital for cellular energy metabolism and biosynthesis 55. By modulating succinyl-CoA levels, tumor cells can adjust their metabolic pathways to meet their demands for energy and biomacromolecules. Succinyl-CoA ligase (SUCL) converts succinyl-CoA in the TCA cycle into succinate. Succinyl-CoA ligase GDP-forming subunit β (SUCLG2) is a key component of the SUCL enzyme complex. SIRT5 acts on SUCLG2, desuccinylating SUCLG2 at K93, leading to the ubiquitination and degradation of SUCLG2. The decreased level of SUCLG2 can upregulate the succinylation level of mitochondrial proteins and inhibit the function of key metabolic enzymes by reducing enzyme activity or protein stability, thereby inhibiting the mitochondrial function of LUAD cells 56. In the TCA cycle, isocitrate dehydrogenase (IDH) catalyzes the oxidative decarboxylation of isocitrate to generate α-ketoglutarate, which, in turn, activates SDH. SDH participates in both the TCA cycle and the electron transport chain, and its activation and consumption of succinate enhance the TCA cycle, thereby promoting mitochondrial respiration 54. However, IDH mutations produce the oncogenic metabolite R-2-hydroxyglutarate (R-2HG), which can competitively inhibit SDH and induce mitochondrial hyper-succinylation, resulting in altered cancer metabolism and apoptosis resistance 41. Studies have also found lysine succinylation can directly regulate the TCA cycle by activating key metabolic enzymes. SIRT5 can desuccinylate the key TCA metabolic enzyme citrate synthase(CS) at K393 and K395, enhancing its activity and promoting colon cancer cell proliferation and migration 57.

Moreover, lysine succinylation can promote the biosynthesis of nucleotides and DNA replication in tumor cells by regulating the final product of the PPP, ribose-5-phosphate 58. Glucose-6-phosphate dehydrogenase (G6PD) and transketolase (TK) are the key catalytic enzymes in the PPP pathway 59, 60. Studies have shown that the succinylation level of G6PD is upregulated in lung cancer cells 61. Similarly, in breast cancer cells, succinylation of TK can significantly alter its ability to bind to transient multienzyme complexes and affect its metabolic function 62.

#### 3.1.2 Lysine Succinylation in Amino Acid Metabolism

In addition to glucose metabolism, lysine succinylation can also influence tumorigenesis and progression through amino acid metabolism. Amino acids are essential building blocks for cell growth and proliferation, and tumor cells often exhibit abnormal metabolic patterns of amino acids to support their rapid proliferation demands 63.

Glutamine can provide energy for tumor cells by participating in the TCA cycle, while its carbon skeleton can be used for the synthesis of nucleic acids, lipids, and proteins, all of which are essential for the rapid proliferation of tumor cells 64, 65. Furthermore, the nitrogen generated from glutamine metabolism can synthesize glutathione, help maintain the intracellular reduced state, and resist oxidative stress. Certain intermediates of glutamine metabolism can also promote angiogenesis, supporting tumor growth and metastasis 11. Bcl-2-associated athanogene 3 (BAG3) is involved in selective macroautophagy/autophagy, and BAG3 can reduce the expression of SIRT5. This hinders desuccinylation of GLS at K158 and K164 and promotes the stabilization of GLS. It promotes glutamine consumption and glutaminolysis to enhance autophagy and the proliferation of tumor cells 66. Renal-type GLS is highly expressed in pancreatic ductal adenocarcinoma, and under oxidative stress conditions, tumor cells regulate the succinylation and enzymatic activity of GLS through succinyl-CoA synthetase ADP-forming subunit β (SUCLA2). This can promote glutamine metabolism and enhance the survival and proliferation of pancreatic ductal adenocarcinoma tumor cells 67.

Serine, as a donor of one-carbon units, participates in nucleic acid synthesis and methylation reactions, and its importance in tumor metabolism cannot be overlooked. Serine hydroxymethyltransferase 2 (SHMT2) converts serine into glycine and a one-carbon unit bound to tetrahydrofolate, supporting the synthesis of thymidines and purines, thereby promoting tumor growth 68. SIRT5 acts on SHMT2 at K280, desuccinylating it. This accelerates serine catabolism, which, in turn, increases the production of purines and NADPH by providing more one-carbon units, thereby accelerating the proliferation of tumor cells 69.

#### 3.1.3 Lysine Succinylation in Lipid Metabolism

Lipid metabolism is a vital source and storage mode of intracellular energy, and it constitutes an essential component of cell membranes. Disruptions in lipid metabolism frequently accompany the development and progression of tumors. ACOX1, a pivotal rate-limiting enzyme in peroxisomes during the β-oxidation process of fatty acids, undergoes activity regulation through SIRT5-mediated desuccinylation. This process, by inhibiting the formation of ACOX1 dimers to inhibit its activity, not only maintains a balance of fatty acid oxidation but also promotes the homeostatic generation of H_2_O_2_, thereby inhibiting the progression of liver cancer 33. Sterol response element binding protein 1a (SREBP1a), a transcription factor important for lipid synthesis, activates genes implicated in the entire lipid synthesis program 70. SIRT7 facilitates the desuccinylation of PRMT5 at K387 within cells. Consequently, this enhances the methyltransferase activity of PRMT5, resulting in the methylation of SREBP1a. This, in turn, elevates the biosynthesis levels of cholesterol, fatty acids, and triglycerides within cells 36.

### 3.2 Regulation of Gene Expression by Lysine Succinylation

**Figure 3:**
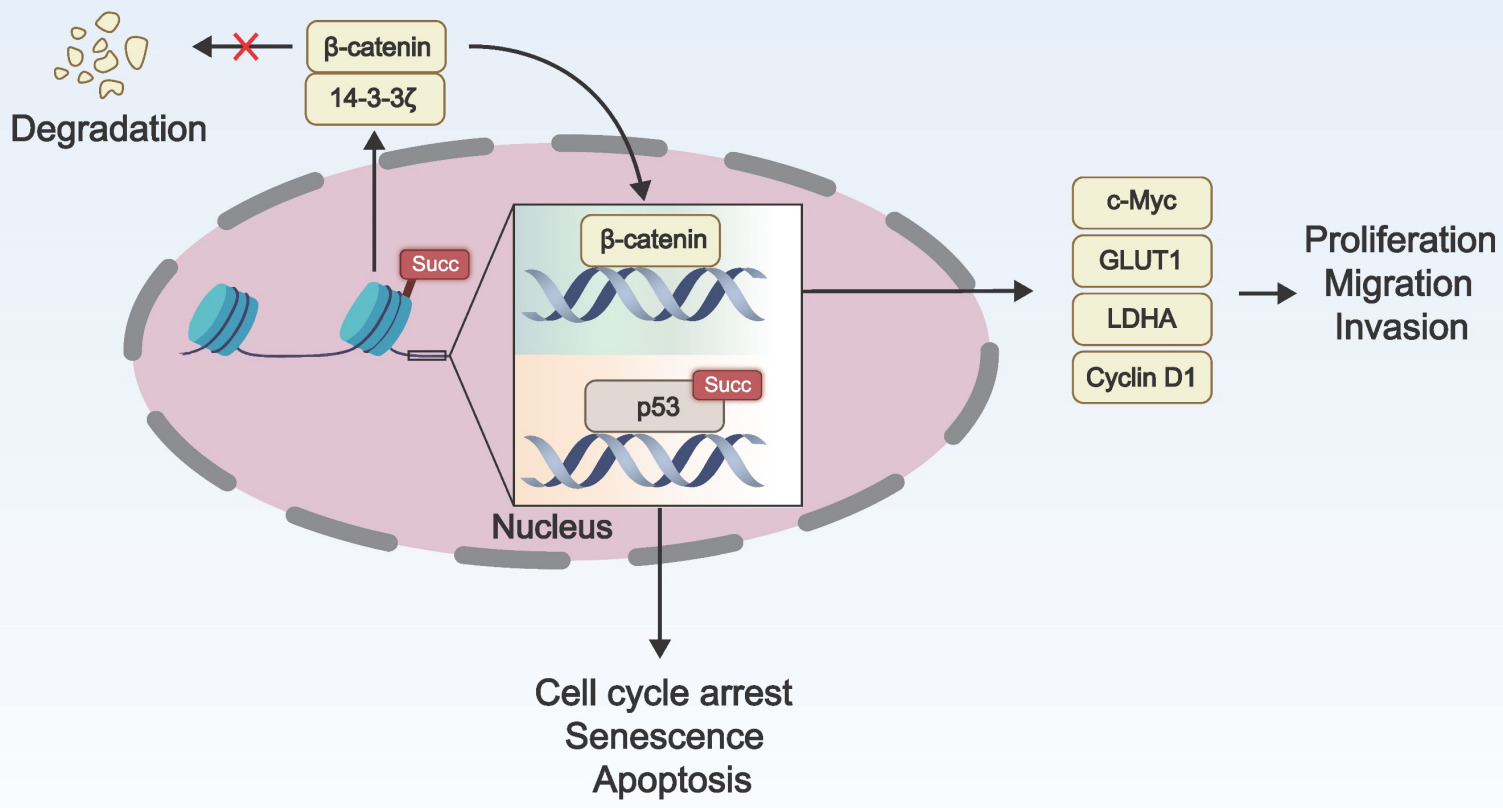
** The role of lysine succinylation in gene expression.** Succinylation of histone H3 enhances the expression of 14-3-3ζ by regulating histone structure and inhibits the degradation of β-catenin, which in turn promotes the expression of cyclin D1, cellular myelocytomatosis oncogene (c-Myc), glucose transporter type 1 (GLUT1), and lactate dehydrogenase A (LDHA). This process facilitates the proliferation, invasion, and migration of cancer cells. The succinylation of p53 enhances its transcriptional activity, leading to cell cycle arrest, senescence, and apoptosis.

Lysine succinylation of histones can modify the interaction with DNA, thereby affecting the proportion of heterochromatin and euchromatin. The succinylation of transcription factors promotes tumor growth and carcinogenesis by affecting their nuclear localization, transcriptional activity, and stability. Therefore, lysine succinylation can regulate gene expression by altering histone structure or modulating the activity of transcription factors, impacting the occurrence and development of tumors (**Figure 3**).

Succinylation modification of histones enables the regulation of gene expression without altering the DNA sequence 71. Shahidian and coworkers discovered that succinylation of H3 at K122 affected the structural stability of nucleosomes and facilitated the binding of transcription factors, leading to transcriptional activation and maintenance of transcriptional activity, ultimately promoting gene expression 25. Tong *et al.* found that KAT2A regulated the succinylation of histone H3 at K79 in the promoter region of YWHAZ (encoding 14-3-3ζ), enhancing 14-3-3ζ expression. This prevented the degradation of the oncogenic protein β-catenin and promotes the expression of cyclin D1, c-Myc, GLUT1, and LDHA, ultimately contributing to the proliferation, migration, and invasion of pancreatic ductal adenocarcinoma cells 14.

Succinylation modification may potentiate the nuclear translocation of transcription factors, enabling them to bind and activate the transcription of target genes more effectively. P53, a prototypical tumor suppressor protein and transcription factor, triggers cell cycle arrest, senescence, and apoptosis in response to DNA damage, excessive proliferation signals, hypoxia, nutrient deprivation, and other stress processes, thereby maintaining genomic stability 72. Liu and coworkers discovered that SIRT5 mediated desuccinylation of p53 at K120 and inhibited its transcriptional activity, subsequently suppressing the expression of p53 target genes and p53-mediated apoptosis, ultimately contributing to tumorigenesis 73.

It is evident that histone succinylation modification plays a pivotal role in gene expression regulation by modulating nucleosome stability, transcription factor binding capacity, and transcriptional activity. Meanwhile, transcription factors can promote the expression of specific genes, such as those related to cell proliferation, and they can also inhibit the expression of apoptosis-related genes by regulating transcription factors like p53, thereby influencing tumorigenesis.

### 3.3 Influence of Lysine Succinylation on Signal Transduction

**Figure 4:**
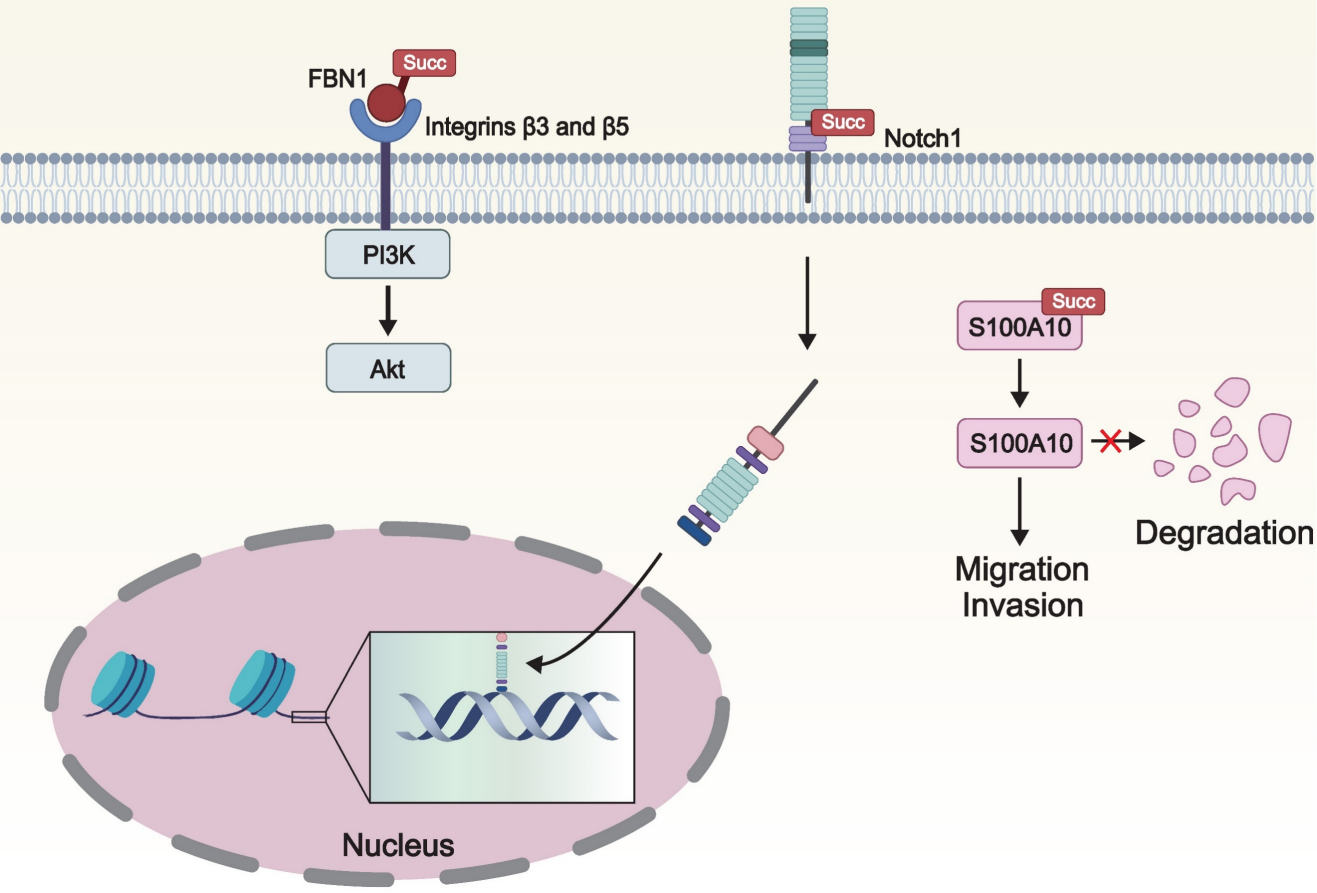
** The impact of lysine succinylation on signal transduction.** The succinylation of FBN1 activates the intracellular PI3K/Akt signaling pathway, contributing to the progression of cancer cells. Notch1 is cleaved when it is succinylated, resulting the release of Notch intracellular domain, which stimulates the target gene expression. Succylation of S100A10 inhibits its ubiquitination and subsequent proteasomal degradation, thereby promoting the invasion and migration of cancer cells. FBN1: fibrillin 1; PI3K: phosphatidylinositol-3 kinase; Akt: protein kinase B; S100A10: S100 calcium-binding protein A10.

Lysine succinylation regulates signaling pathways by altering the structure or function of critical signaling proteins, thereby playing a significant role in biological processes such as tumor cell growth, proliferation, migration, and metastasis (**Figure 4**). Aberrant activation of the PI3K/Akt signaling pathway is closely associated with proliferation, survival, invasion, and metastasis in various types of tumors 74. Among them, phosphatidylinositol-3 kinase (PI3K) generates the critical lipid second messenger phosphatidylinositol 3,4,5-trisphosphate (PIP3), which activates protein kinase B (Akt). This cascade subsequently promotes cell cycle progression, inhibits apoptosis, enhances angiogenesis, and promotes the invasion and metastasis of tumor cells 75. Wang and team discovered that succinylation of fibrillin 1 (FBN1) promoted the long-term accumulation and deposition of FBN1 and activated the intracellular PI3K/Akt signaling pathway, contributing to the progression of gastric cancer 76.

S100 signaling molecules constitute a family of calcium-binding cytoplasmic proteins that participate in calcium signaling and various oncogenic signaling pathways, playing a pivotal role in tumor migration and invasion 77. Wang and colleagues found that S100 calcium-binding protein A10 (S100A10), a member of the S100 protein family, is overexpressed in gastric cancer. Furthermore, CPT1A causes the succinylation of S100A10 at K47, which inhibits ubiquitination and subsequent proteasomal degradation of S100A10, thereby promoting the invasion and migration of gastric cancer 78.

Notch protein is a crucial signaling and embryonic development regulatory receptor protein. Notch1, as a receptor on the cell surface, upon activation, releases its intracellular domain, which then acts as a transcription factor and enters the nucleus to regulate cellular processes such as differentiation, apoptosis, and proliferation 79. Dysregulation of the Notch pathway is closely associated with the occurrence of malignant tumors 80. KAT2A mediated the succinylation of Notch1 at K2177, enhancing its activity, thereby activating the Notch pathway and promoting the proliferation and differentiation of stem cells 81.

We can discern that the aberrant regulation of PI3K/Akt, Notch, and S100 signaling pathways is intimately tied to tumor progression. In particular, by modulating key molecules within these pathways, succinylation modification facilitates tumor cell proliferation, survival, invasion, metastasis, and the ability to adapt to hypoxic environments.

### 3.4 Influence of Lysine Succinylation on Immune Microenvironment

**Figure 5:**
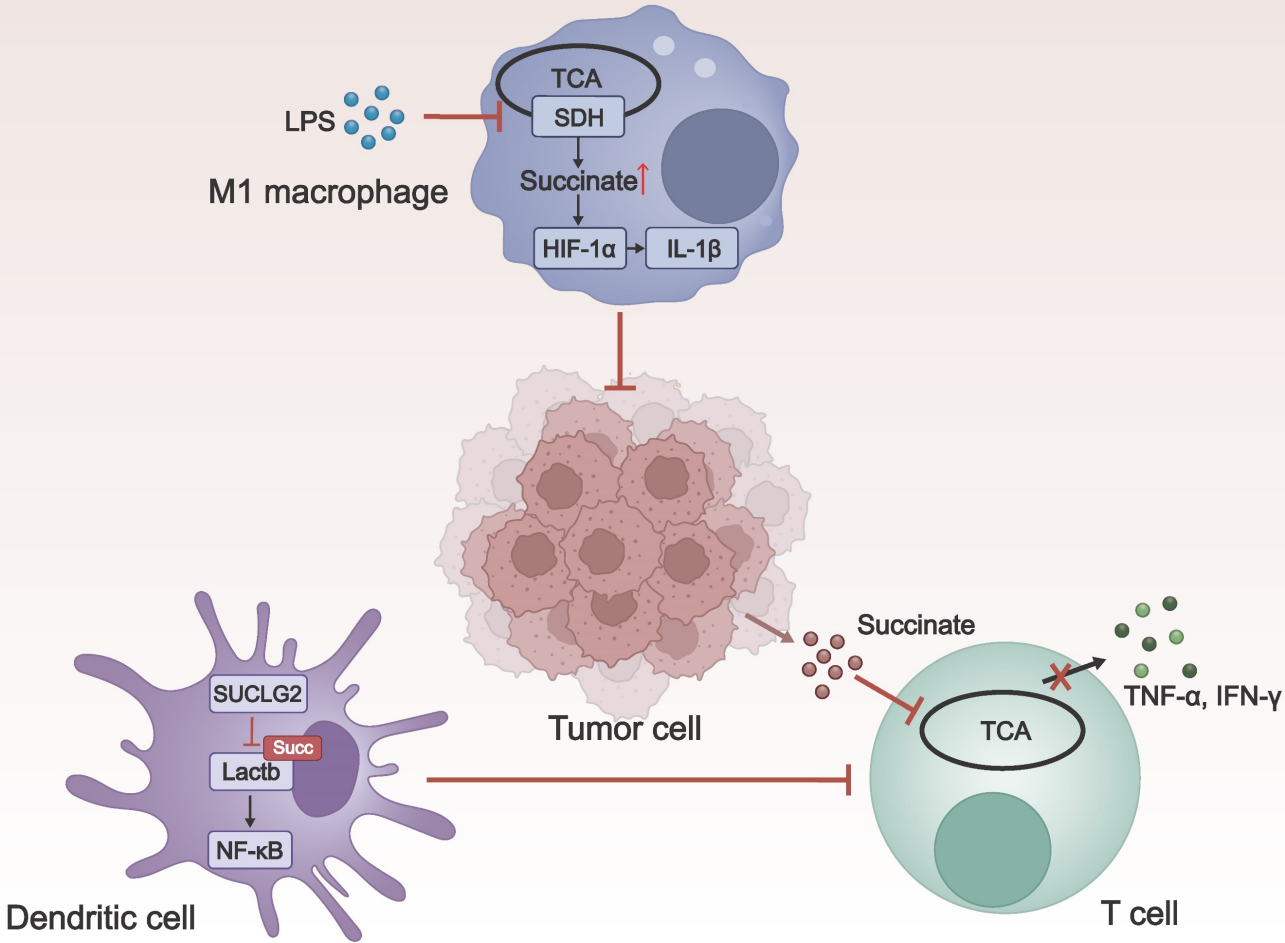
** The influence of lysine succinylation on the immune microenvironment.** LPS-induced succinate accumulation induces succinylation and promotes the production of HIF-1α, which, in turn, induces the production of IL-1β, promoting the activation of macrophages to the M1 type, and enhancing their anti-tumor effects. In dendritic cells, SUCLG2 inhibits the succinylation of Lactb, thereby preventing the activation of NF-κB signaling, which suppresses T cells activation and leads to immune tolerance. The uptake of tumor-associated succinate by T cells significantly inhibits T cell degranulation and the production of IFN-γ and TNF-α, resulting in impaired effector functions of T cells. LPS: lipopolysaccharide; TCA: tricarboxylic acid; SDH: succinate dehydrogenase; IL-1β: interleukin-1beta; SUCLG2: succinyl-CoA ligase GDP-forming subunit β; Lactb: serine β-lactamase-like protein; NF-κB: nuclear factor κB; TNF-α: Tumor Necrosis Factor-α; IFN-γ: interferon-γ.

Tumor cells exist within an immune microenvironment composed of various immune cells, including macrophages, dendritic cells, T cells, and others. This microenvironment is crucial in constraining tumor growth and dissemination 82. As an essential PTM, lysine succinylation can regulate the function of immune cells through signaling molecules such as succinate (**Figure 5**). Delving into the role of lysine succylation in the immune microenvironment is vital for developing novel anti-cancer drugs.

The role of macrophages in the tumor microenvironment is multifaceted. Depending on different activators, macrophages can polarize in two different directions. Macrophages induced by lipopolysaccharide (LPS) (M1/M (LPS)) promote tumor regression, while those induced by IL-4 (M2/M (IL-4)) facilitate tumorigenesis 83. Following LPS stimulation, SDH activity was inhibited, leading to the accumulation of succinate and an increase in lysine succinylation. This, in turn, stabilizes downstream hypoxia-inducible factor 1α (HIF-1α), which induces the production of the crucial proinflammatory cytokine interleukin-1β (IL-1β) and influences M1 macrophage polarization, endowing them with the ability to kill tumor cells 84, 85.

Dendritic cells play a critical role in initiating and regulating the immune response. Zhang *et al.* discovered the key metabolic enzyme SUCLG2 in the mitochondrial TCA cycle, as well as Lactb, a mitochondrial protein that positively regulates nuclear factor κB (NF-κB) signal transduction. They proposed that SUCLG2 prevented the activation of NF-κB signaling by inhibiting the succinylation of Lactb at K288. This helps to maintain the tolerogenic phenotype of regulatory dendritic cells, promoting tumor evasion of immune surveillance 86.

T cells can recognize and attack tumor cells through immune surveillance. However, the tumor microenvironment (TME) is highly immunosuppressive, capable of influencing T cell function through various mechanisms, leading to T cell exhaustion or inactivation 87. As a substrate for succinyl-CoA, succinate can promote protein succinylation, affecting cellular functions. Gudgeon and colleagues found that the uptake of tumor-associated succinate significantly inhibited T cell degranulation, interferon-γ (IFN-γ) expression, and Tumor Necrosis Factor-α (TNF-α) expression, resulting in impaired effector functions of T cells 88.

Taken together, protein succinylation modification plays a double-edged role in the immune microenvironment. On the one hand, it can contribute to anti-tumor immunity by activating host defensive immune cells; on the other hand, it promotes the immune escape of tumor cells by inhibiting the killing behavior of immune cells.

## 4. Anti-cancer Agents Modifying Protein Succinylation/Desuccinylation

The in-depth study of lysine succinylation provides a theoretical basis for the development of novel targeted therapeutic strategies. Currently, a variety of selective and highly efficient succinylation inhibitors and activators have been discovered as anti-cancer agents. Modulating the lysine succinylation level can affect tumor cell metabolism, proliferation, survival, and immune response. We summarize the currently evaluated anti-cancer agents modifying lysine succinylation in **Table 2**.

Abril and coworkers developed a small molecule inhibitor of SIRT5 called DK1-04e that inhibited the metastatic properties of breast cancer cells through desuccinylation of various metabolic enzymes, e.g., IDH, GLS, SHMT2, and PKM2, and significantly impaired tumor growth in experimental mice 89. Polletta and colleagues found that another SIRT5 inhibitor, MC3482, through modifying ε-N-glutamyl lysine, inhibited succinylation of GLS, thereby promoting autophagy in breast cancer cells and inhibiting tumor initiation and progression 90. When bladder cancer cells were treated with the HSP90 inhibitor AUY922, H4K20succ and H3K122succ were identified, suggesting that HSP90 inhibitors may exert anti-tumor effects through lysine succinylation 91. Qi and coworkers developed a small molecule, Compound 8, which can block succinylation-mediated mitochondrial translocation of PKM2 and inhibit tumor development 51.

In addition to the aforementioned development of new agents, certain clinically used medications have also been found to modulate protein succinylation to inhibit tumor growth. It is well known that aspirin holds significant potential in cancer prevention and treatment 92. Among these, the findings from Wang's team indicate that aspirin inhibited PGAM1 activity by attenuating its succinylation in liver cancer cells, thereby restricting the glycolytic process 17. Furthermore, Zhu and colleagues observed an upregulation of 179 succinylation sites in colon cancer HCT116 cells treated with dichloroacetate (DCA), a pyruvate dehydrogenase kinase inhibitor. This suggests that DCA can induce lysine succinylation, facilitating pyruvate transport to the mitochondrion. This, in turn, enhances mitochondrial glucose oxidation, leads to mitochondrial depolarization, and ultimately restores cell membrane potential to normal levels, thereby exerting its anticancer effects 93. Additionally, lovastatin, a naturally occurring lipophilic statin, has garnered increasing attention for its antitumor effects 94. We found that lovastatin can induce lysine succinylation of cytoskeleton-related proteins, inhibiting EMT and metastasis of triple-negative breast cancer stem cells both *in vitro* and *in vivo* 95.

Although anti-cancer agents specifically modifying lysine succinylation are still in the preclinical phase, they have already shed light on the potential of improving cancer outcomes. In the future, developing more precise and effective succinylation-targeted therapeutic drugs will offer a promising treatment option for cancer patients.

## 5. Concluding Remarks and Prospects

As a newly discovered PTM, lysine succinylation has been shown to have a non-negligible connection with the development and occurrence of various tumors. This article summarizes the regulatory enzymes of lysine succinylation and discusses lysine succinylation from four aspects: metabolic reprogramming, regulation of gene expression, signal transduction, and the immune microenvironment. Finally, this article discusses the current research on drugs targeting protein succinylation. Hopefully, the study on the involvement of lysine succinylation in cancer will shed light on elucidation of cancer pathogenesis and discovery of novel strategies for cancer therapy.

Future research should delve deeper into the specific mechanisms of lysine succinylation in different tumor types and its intricate interplay with other PTMs. Additionally, the fact that numerous succinylases and desuccinylases target multiple lysine modifications underscores the complexity of crosstalk, where different lysine modifications may exert antagonistic effects on substrate functions. Therefore, precisely regulating these enzymes to coordinate their interactions with other lysine modifications, aiming to enhance anti-tumor effects jointly, poses a pivotal scientific challenge in the current research. Simultaneously, a profound understanding of the role of lysine succinylation in the tumor microenvironment will facilitate the development of novel tumor immunotherapy strategies. These studies will bring breakthrough advancements in cancer treatment, potentially leading to substantial improvements in cancer patients' survival rates and quality of life.

## Figures and Tables

**Table 1:** Writers, earsers, and reader of lysine succinylation in cancer

Category	Enzyme	Mechanism	Fuction	Reference
Writer	α-KGDHC	Increasing the succinylation of H3 at K79	Promoting glioma cell proliferation	11,12
	KAT2A	Increasing the succinylation of H3 at K79	Promoting proliferation, migration, and invasion of glioma and PDAC cell	11,14
	HAT1	Succinylating histone H3 at K122 and PGAM1 at K99	Promoting HCC, CCA, and PC progression	16
	CPT1A	Succinylating LDHA at K222 and inhibiting its degradation	Promoting proliferation, invasion, and migration of GC cell	19
		Promoting succinylation of MFF at K302	Promoting growth and proliferation of OC cell	20
	OXCT1	Increasing succinylation of Latcb at K284	Promoting HCC progression	23
	P300	Succinylating H3 at K122	Promoting BC cell proliferation	25
		Succinylating pivotal cytoplasmic glycolytic enzymes (e.g., PGK1, PKM, PFKL, and ALDOA)	Promoting malignant progression of lung cancer	26
Eraser	SIRT5	Mediating the desuccinylation of GLS at K158	Supporting proliferation and tumorigenesis in BC	32
		Desuccinylating SDHA at K547	Promoting ccRCC tumorigenesis	10
		Mediating ACOX1 desuccinylation	Inhibiting oxidative stress and suppress HCC development	33
		Inhibiting the succinylation of OGDH	Downregulating cells growth and migration of GC	34
		Desuccinylating and activating SOD1	Inhibiting LC cell growth	35
	SIRT7	Mediating desuccinylation of PRMT5 at K387	Promoting tumour growth and metastasis of BC and HCC	36
Reader	GAS41	Recognizing the succinylation of H3 at K122 through a pH-dependent manner	Promoting the development of NSCLC	43,44

α-KGDHC: α-ketoglutarate dehydrogenase complex; KAT2A: lysine acetyltransferase 2A; HAT1: histone acetyltransferase 1; CPT1A: carnitine palmitoyltransferase 1A; OXCT1: 3-oxoacid CoA-transferase 1; SIRT5: sirtuin 5; SIRT7: sirtuin 7; GAS41: glioma amplified sequence 41; PGAM1: phosphoglycerate mutase 1; LDHA: lactate dehydrogenase A; MFF: mitochondrial fission factor; Lactb: serine β-lactamase-like protein; PGK1: phosphoglycerate kinase 1; PKM2: pyruvate kinase M2; PFKL: phosphofructokinase 1; ALDOA: fructose-bisphosphate aldolase A; GLS: glutaminase; SDHA: succinate dehydrogenase complex subunit A; ACOX1: acyl-CoA oxidase 1; OGDH: 2-oxoglutarate dehydrogenase; SOD1: copper/zinc superoxide dismutase; PRMT5: protein arginine methyltransferase 5; PDAC: pancreatic ductal adenocarcinoma; HCC: hepatocellular carcinoma; CCA: cholangiocarcinoma; PC: pancreatic cancer; GC: gastric cancer; OC: ovarian cancer; BC: breast cancer; ccrCC: clear cell renal cell carcinoma; LC: lung cancer; NSCL: non-small cell lung cancer

**Table 2:** Anti-cancer agents modifying lysine succinylation/desuccinylation

Agent	Modified protein	Lysine succinylation/desuccinylation	Mechanism	Cancer	Reference
DK1-04e	IDH, GLS, SHMT2, and PKM2	Succinylation	Increasing the global protein succinylation and inhibiting tumor growth.	Breast cancer	87
MC3482	GLS	Succinylation	Increasing the succinylation of GLS, thereby enhancing ammonia-induced autophagy and inhibiting tumor initiation.	Breast cancer	88
AUY922	Histone	Succinylation	Exerting antitumor effects through histone succinylation, including succinylation of H4 at K20 and H3 at K122.	Bladder cancer	89
Compound 8	PKM2	Desuccinylation	Blocking PKM2 mitochondrial translocation, might through attenuating its succinylation at K433, and thus inhibiting tumor growth.	Colon cancer	49
Aspirin	PGAM1	Desuccinylation	Inhibiting PCAM1 activity by attenuating its succinylation in liver cancer cells, thereby restricting the glycolytic process.	Liver cancer	17
Dichloroacetate	/	Succinylation/desuccinylation	Playing a significant role in protein synthesis and cellular energy metabolism.	Colon cancer	91
Lovastatin	Cytoskeletal-associated proteins	Succinylation/desuccinylation	Inducing lysine succinylation of cytoskeleton-associated proteins, and inhibiting EMT and metastasis of TNBC CSCs.	Triple-negative breast cancer	93

IDH: isocitrate dehydrogenase; GLS: glutaminase; SHMT2: serine hydroxymethyltransferase 2; PKM2: pyruvate kinase M2; PGAM1: phosphoglycerate mutase 1

## Abbreviations

ACOX1: acyl-CoA oxidase 1
Akt: protein kinase B
α-KGDHC: α-ketoglutarate dehydrogenase complex
BAG3: bcl-2-associated athanogene 3
CPT1A: carnitine palmitoyltransferase 1A
CS: citrate synthase
DCA: dichloroacetate
EMT: epithelial-to-mesenchymal transition
FBN1: fibrillin 1
G6PD: glucose-6-phosphate dehydrogenase
GAS41: glioma amplified sequence 41
GLS: glutaminase
HAT1: histone acetyltransferase 1
HBV: hepatitis B virus
HDAC1/2/3: histone deacetylase1/2/3
HIF-1α: hypoxia-inducible factor 1α
IDH: isocitrate dehydrogenase
IFN-γ: interferon-γ
IL-1β: interleukin-1β
KAT2A: lysine acetyltransferase 2A
Lactb: serine β-lactamase-like protein
LDHA: lactate dehydrogenase A
LPS: lipopolysaccharide
LSTase: lysine succinyltransferase
LUAD: lung adenocarcinoma
MFF: mitochondrial fission factor
NF-κB: nuclear factor κB
OGDH: 2-oxoglutarate dehydrogenase
OXCT1: 3-oxoacid CoA-transferase 1
PDH: pyruvate dehydrogenase
PGAM1: phosphoglycerate mutase 1
PGK1: phosphoglycerate kinase 1
PI3K: phosphatidylinositol-3 kinase
PIP3: phosphatidylinositol 3,4,5-trisphosphate
PKM2: pyruvate kinase M2
PPP: pentose phosphate pathway
PRMT5: protein arginine methyltransferase 5
PTMs: post-translational modifications
R-2HG: R-2-hydroxyglutarate
ROS: reactive oxygen species
S100A10: S100 calcium-binding protein A10
SDH: succinate dehydrogenase
SDHA: succinate dehydrogenase complex subunit A
SHMT2: serine hydroxymethyltransferase 2
SIRT5: sirtuin 5
SIRT7: sirtuin 7
SOD1: superoxide dismutase 1
SREBP1a: sterol response element binding protein 1a
succinyl-CoA: succinyl coenzyme A
SUCL: succinyl-CoA ligase
SUCLA2: succinyl-CoA synthetase ADP-forming subunit β
SUCLG2: succinyl-CoA ligase GDP-forming subunit β
TCA: tricarboxylic acid
TK: transketolase
TME: tumor microenvironment
TNF-α: tumor necrosis factor-α
VDAC3: voltage-dependent anion channel 3
